# Supplementary material for: Metagenomic binning with assembly graph embeddings
Source: Bioinformatics. 2022 Aug 16;38(19):4481–7. doi: 10.1093/bioinformatics/btac557 (PMC9525014; doi:10.1093/bioinformatics/btac557)
Supplement: btac557_supplementary_data [file btac557_supplementary_data.zip › amber outputs.zip!/amber outputs/simHC/genome/metabat_contigs/purity_vs_bin_size_simHC+.pdf]

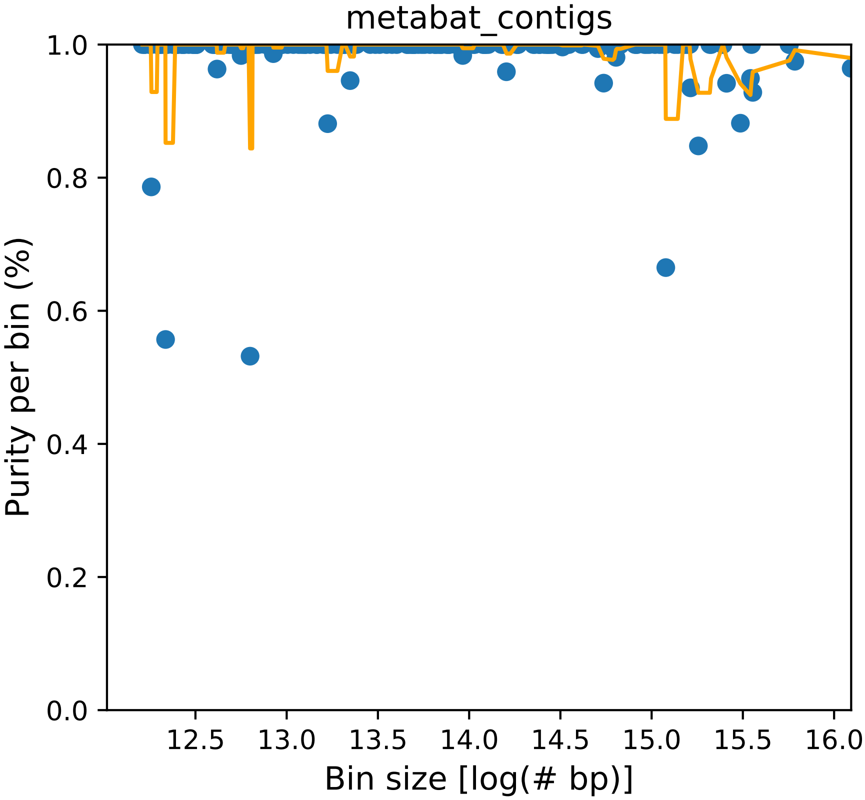
metabat_contigs
Purity per bin (%)
Bin size [log(# bp)]
1.0
0.8
0.6
0.4
0.2
0.0
12.5
13.0
13.5
14.0
14.5
15.0
15.5
16.0
